# PhysIOpathology of NEuromuscular function rElated to fatigue in chronic Renal disease in the elderly (PIONEER): study protocol

**DOI:** 10.1186/s12882-020-01976-6

**Published:** 2020-07-25

**Authors:** Antoine Chatrenet, Bruno Beaune, Antioco Fois, Camille Pouliquen, Jean-Michel Audebrand, Massimo Torreggiani, Damien Paris, Sylvain Durand, Giorgina Barbara Piccoli

**Affiliations:** 1 Nephrology, Centre Hospitalier Le Mans, Le Mans, France; 2 Laboratory “Movement, Interactions, Performance” (EA 4334), Le Mans University, Le Mans, France; 3 Endocrinology and diabetology, Centre Hospitalier Le Mans, Le Mans, France; 4 Department of clinical and biological sciences, University of Torino, Torino, Italy

**Keywords:** Muscle fatigue, Tiredness, Electromyographic features, Pre-dialysis

## Abstract

**Background:**

Chronic Kidney Disease (CKD) is associated with reduced muscular strength resulting in profound fatigue. The physiopathology of these changes, their prevalence and evolution are still debated. Moreover, we have little data on elderly CKD patients. The present study protocol aims to 1) quantify the prevalence of low muscle strength (dynapenia) in a cohort of elderly patients with advanced CKD and to 2) characterize their force production coupled with electromyographic features and the symptoms of fatigue compared to a matched control group.

**Methods:**

This is a case-control, prospective, interventional study. Inclusion criteria: age ≥ 60 years; CKD Stage 3b-5; clinical stability (i.e. no hospitalization and ≤ 25% in creatinine increase in the previous 3 months). Controls with normal kidney function will be matched in terms of age, gender and diabetes mellitus (requisite: estimated glomerular filtration rate ≥ 60 ml/min/1.73m^2^ available in the last 6 months). Exclusion criteria for cases and controls: neuromuscular disease, life expectancy < 3 months.

The handgrip strength protocol is an intermittent test consisting in 6 series of 9 repetitions of 3-s sub-maximum contractions at 40% of the maximum voluntary contraction (MVC) and 2 s of resting time between contractions. Each series is separated by one fast sub-maximum contraction and one MVC. Strength is assessed with a high-frequency handgrip dynamometer paired with surface electromyography. Symptoms of fatigue are assessed using MFI-20 and FACIT-F questionnaires. In order to reach a statistical power of 96%, we plan to enroll 110 subjects in each group.

**Discussion:**

The novelty of this study resides in the application of an already validated set of tests in a population in which this combination (dynamometer, electromyography and questionnaires) has not previously been explored. We expect a high prevalence of dynapenia and a higher fatigability in CKD patients. A positive correlation is expected between reported fatigue and fatigability.

Better appreciation of the prevalence and the relationship between fatigability and a sensation of fatigue can help us target interventions in CKD patients to improve quality of life and survival.

**Trial registration:**

The study was approved by Ethical Committee EST III n°20.03.01 and was recorded as a Clinical Trial (NCT04330807) on April 2, 2020.

## Background

Chronic kidney disease (CKD) is defined as a structural or functional abnormality of the kidneys that has lasted at least 3 months and has implications for health [1]. The kidneys have a major role in the maintenance of homeostasis, ensuring water and electrolyte balance that directly determines the functioning of vital organs. It is for this reason that a decline in kidney function induces a number of systemic metabolic alterations, especially in the most advanced stages i.e., glomerular filtration rate (GFR): ≤45 ml/min/1.73m^2^ [2].

Among the symptoms associated with advanced CKD, fatigue is often reported by patients, with a prevalence ranging between 50 and 70% [3]. Fatigue is defined as a subjective sensation of weakness, increasing sense of effort, mismatch between exerted and actual effort [4]. Fatigue, which has a remarkable impact on a patient’s everyday activities and quality of life, can be subjectively evaluated using validated questionnaires.

The pathogenesis of fatigue is complex. Fatigue can arise from disease-related issues (e.g., diffuse vascular disease, anemia, changes in lifestyle habits), psychological conditions (e.g., stress, depression, sleep disorders) and pathophysiological changes (e.g., decline in aerobic capacity or muscle strength) [5]. These problems are common in other chronic diseases, such as the consequences of stroke [6], cancer [7, 8], multiple sclerosis [9], rheumatoid arthritis [10, 11] and diabetes mellitus [12–15]. In all of them, observed neuromuscular changes are associated with fatigue [16].

Neuromuscular fatigability is defined as a reduction in the patient’s muscular ability to perform a standardized task [17]. In general, inability to exert the desired force is not restricted to muscle-mass decline. Several factors concur to determine muscle function: fiber type (i.e., slow or fast twitches), metabolism (i.e., aerobic or anaerobic), presence of fat infiltration or fibrosis and interactions at the neuromuscular junction [18]. CKD induces chronic muscle function changes [19–21]: this is relatively well documented for dialysis patients [22, 23], but we still have little data about the loss of muscle strength (dynapenia [24]) in CKD patients.

In the course of chronic dialysis, the loss of muscle function is worsened by several factors, such as acidosis, loss of amino acids (up to 12 g per hemodialysis session or 4 to 6 g per day on peritoneal dialysis) and/or the presence of anorexia. In this context, the loss of strength is mainly attributable to a decrease in muscle-mass, characterized by the loss, or a decrease in size, of the contractile units [25–27]. Conversely, changes in the excitation-contraction coupling system have been little explored and are poorly understood, especially in patients with advanced CKD.

The grip isometric analysis (i.e., handgrip) is the most commonly used test to assess strength in patients who have advanced CKD [28]. The handgrip test is simple and rapidly performed; it yields reproducible results and can easily be integrated into routine clinical practice. In CKD patients, this test demonstrates a close correlation with survival, nutritional status and quality of life [29–33]. Moreover, an isometric contraction is less traumatic than a dynamic contraction [34]. Finally, a handgrip is a simple routine gesture and does not usually cause pain.

Thus, the aim of the study is to assess the neuromuscular features of elderly patients with advanced CKD and symptoms of fatigue coupled with dynapenia. To our knowledge, this is the first study to apply a complex set of validated tests to a large population of elderly patients who have advanced CKD but are not on dialysis, with respect to an age, sex and diabetes matched control group. The research will highlight neuromuscular features associated with dynapenia in elderly CKD patients. In addition, the relationship between dynapenia and subjective fatigue will be analyzed to identify novel therapeutic interventions. These findings will be rapidly translated into clinical practice, with a view to adapting physical activity programs to improve neuromuscular function in the management of CKD symptoms.

## Methods

### Aims

The primary objective of this trial is to define the prevalence of dynapenia in elderly CKD Stage 3b-5 patients. This will be achieved through the analysis of handgrip strength.

The secondary objective is to define the characteristics of dynapenia in elderly patients with advanced CKD during a standardized task considering:

- the evolution of MVC;
- the evolution of the discharge frequency of motor units;
- motor-unit recruitment ability;
- the evolution of the delay between the nervous stimulus and contraction;
- the relationship between dynapenia and subjective fatigue.

### Clinical implications

By shedding light on the specific characteristics of dynapenia and its link with a patient’s sense of fatigue and fatigability [4], the PIONEER study intends to guide the management of adapted physical activities recommended by academic associations.

### Study design

A case-control, interventional, prospective study.

### Setting of study

**Fig. 1:**
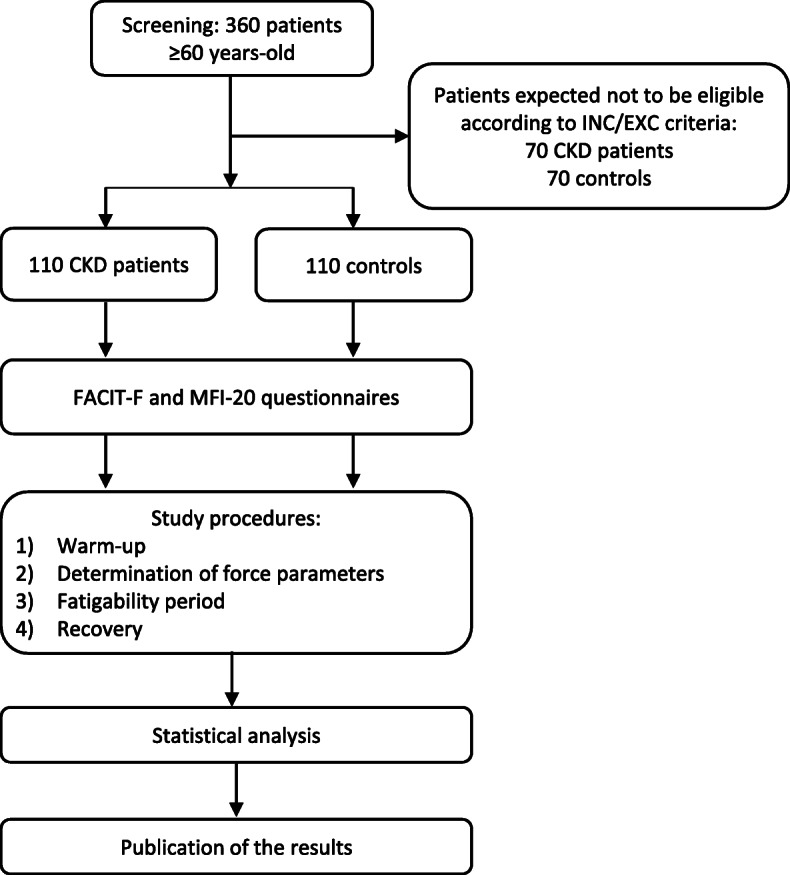
Flowchart of the study. INC: inclusion criteria; EXC: exclusion criteria

The study will be carried out in Le Mans hospital (France), in the outpatient unit for the care of advanced kidney disease (UIRAV, Unité pour l’Insuffisance Rénale chronique AVancée). The UIRAV unit follows about 210 patients which are not on dialysis (90% over the age of 60), whose mean age is 74 years; 60% are males and diabetes mellitus prevalence is approximately 50%. Of these, 180 patients are followed for CKD Stage 3b-5. As previously described by Fois et al. [35], UIRAV’s main objective is to offer frequent follow-up and to delay the start of dialysis. The frequency of controls is based on CKD stage and overall clinical stability. For example, patients with stable kidney disease Stage 3b are seen every 8 to 12 weeks, while patients with CKD Stage 5 are seen at least once a month. Study proceedings is described in Fig. 1.

### Definition and measures

#### Dynapenia

Dynapenia is an age-associated loss of muscle strength that is not caused by neurologic or muscle diseases [24]. In our study, dynapenia will be diagnosed if the mean of three MVC after a brief warm up, is lower than the reference value matched for age and sex. The study by Ramírez-Vélez et al. [36] will provide reference values for elderly patients.

#### Fatigability

**Fig. 2:**
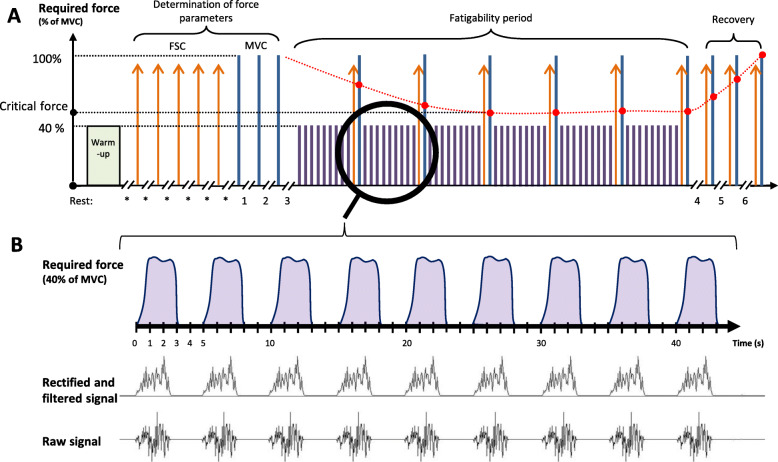
Fatigability protocol with handgrip and EMG acquisition. **a** Neuromuscular fatigability protocol: the green box represents the warm-up period, lasting a maximum of 1.5 min; the orange arrows are FSC; the blue lines are MVC and the purple lines are contractions at 40% of MVC. The red points represent the maximal forces awaited allowing critical force determination, while the evolution is represented with a red spotted line. Rest duration is 20 s (*), 2 min (nos. 1 and 2), 5 min (no. 3), 1 min (no. 4), 2 min (no. 5) and 3 min (no. 6). **b** Parallels between the 40% MVC contractions required and the muscle activation signals recorded. FSC: fast sub-maximum contraction; MVC: maximum voluntary contraction

Fatigability is defined as a reduction in the patient’s muscle performance on a standardized task [17]. Fatigability, which is an indicator of objective fatigue, is evaluated by measuring critical force (F_crit_) during a standardized task. The F_crit_ value corresponds to the asymptotic value of the last stable part of the MVC evolution curve (Fig. 2) [37]. F_crit_ is the maximum exercise intensity that a subject can produce in a given metabolic environment, in other words, it identifies the threshold at which fatigability develops [38].

#### Fatigue

The perception of fatigue is defined as a subjective sensation of weariness, increasing sense of effort, mismatch between expended and actual effort [4]. We will use two questionnaires to assess fatigue: the Functional Assessment of Chronic Illness Therapy-Fatigue (FACIT-F) and the Multidimensional Fatigue Inventory (MFI-20).

FACIT-F is a brief validated French-language questionnaire [39] consisting in 13 simple pragmatic statements (e.g., I am too tired to eat) accompanied by the Likert scale (0: “never or almost never” to 4: “always or almost always”). The final score ranges from 0 to 52, with an inverse relationship between the score and fatigue.

MFI-20 is also a validated French-language version of a widely-used questionnaire [40], focusing on fatigue. It is composed of statements covering self-perception, ranging from extremely positive (e.g., physically I feel/I am in excellent condition) to extremely negative (e.g., physically I feel/I am able to do very little). In the case of the MFI-20, there is a direct relationship between the patient’s score and perceived fatigue while exploring 4 topics: general fatigue, mental fatigue, reduced activity and motivation.

#### Anthropometric, clinical, nutritional and biochemical data

Relevant data will be collected from medical records. They will include age, body mass index, gender, presence of diabetes mellitus (defined as glycemia > 126 mg/dl while fasting or using oral hypoglycemic medications or insulin) and kidney disease (i.e., cause and creatinine, urea and proteinuria values), time of follow-up, Charlson Comorbidity Index (CCI) [41], Malnutrition Inflammation Score (MIS) [42], albumin, total cholesterol, high-density lipoprotein, low-density lipoprotein, triglycerides, uric acid, calcium, phosphorus, parathyroid-hormone, vitamin D, bicarbonate, sodium and potassium values. The most recent available data will be recorded within 3 months for CKD patients and 6 months for control volunteers.

### Inclusion criteria

#### Cases

- Age ≥ 60
- Estimated glomerular filtration rate based on the Chronic Kidney Disease Epidemiology Collaboration (CKD-EPI) equation ≤45 ml/min/1.73m^2^ for at least 3 months
- Stable clinical condition: no hospitalization; serum creatinine increased by a maximum of 25% in the previous 3 months

#### Controls

- Age ≥ 60
- Available blood tests within 6 months showing normal renal function (estimated GFR > 60 ml/min/1.73m^2^) and glycated hemoglobin values for diabetic controls
- Stable clinical condition: no hospitalization in the previous 6 months

### Exclusion criteria

- Inability to give informed consent, if under guardianship or a minor
- Neuromuscular disease
- Dementia
- History of upper limb surgery or pathologies that would make it impossible to fit electromyography (EMG) electrodes and measure handgrip force
- Estimated life expectancy of less than 3 months
- Hospitalization in the 3 months prior to the test
- Participation in another clinical interventional trial
- Acute kidney disease
- Waitlisted for renal replacement therapy or expected to start dialysis within 3 months
- History or evidence of any other clinically significant disorder, condition or disease that in the investigator’s opinion could pose a risk to a participant’s safety or interfere with the study’s evaluation, procedures or completion

### Inclusion modalities

Participants will be informed about the study with a poster displayed in hospital or during medical consultations. Elderly CKD patients will be enrolled via the UIRAV unit. Control subjects will be enrolled through endocrinology, diabetology and dermatology units or during outpatient consultations with patients’ escorts.

### Materials

The handgrip and EMG evaluations will be synchronized using Labview v19 software (National Instruments Corp., Austin TX, USA) that makes it possible to simultaneously display feedback on strength and EMG activity. The handgrip dynamometer used will be the K-Force GRIP (K-Invent Inc., Montpellier, France), with 1000 Hz acquisition frequency and an accuracy of 100 g. The EMG signal will be recorded using the Trigno Wireless Biofeedback System (DelSys Inc., Boston MA, USA) with the Trigno Avanti™ electrodes, which are composed of a 4 silver bar contacts, 10 mm interelectrode, a 1926 Hz acquisition frequency per channel and with a second-order band-pass Butterworth filter set at 20–450 Hz and a second-order low-pass filter set at 100 Hz.

### Intervention

**Fig. 3:**
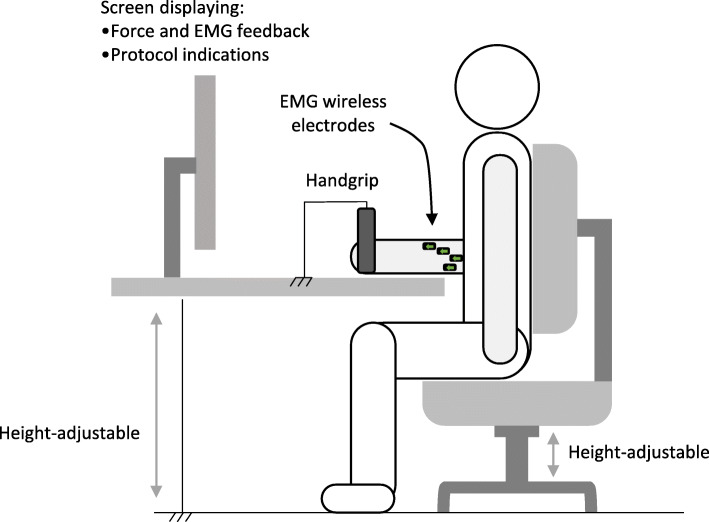
Set-up of the study and standardized position

The patient being tested will be seated, with his back in a straight position, and elbow bent at 90° close to his chest. The patient’s humerus will be vertical and his forearm will be parallel to a height-adjustable support (Fig. 3). The patient’s dominant arm will be chosen.

Preparing the arm for placement of the electrodes will include shaving the skin and cleaning the area hosting the four recording electrodes [43, 44] with a sponge and alcoholic disinfectant to reduce impedance. Electrodes will be placed over the flexor digitorum superficialis muscle belly along the fiber direction while following international SENIAM recommendations and the four electrode positions previously described [43–45].

The exercise, illustrated in Fig. 2, will begin with a warm-up, to familiarize the person with the instruments and situation, at a slow and comfortable pace not exceeding 1 min and 30 sec. The warm-up will consist of dynamic extensions of the hand and fingers: an extension at maximum speed, and a slow and constant flexion of the wrist and fingers. Dynamic stretches have been shown to prepare the nervous system, increase muscle temperature and improve the sensitivity of the connecting bridges between actin and myosin [46] compared to passive stretches, which can be somewhat deleterious in terms of muscle performance [47].

According to the recommendations of Maffiuletti et al. [48], fast sub-maximum contractions (FSC) should be separated from MVC to limit central inhibition. Thus, to determine the baseline FSC, five contractions at maximum speed will be performed with a twenty-second rest interval between them. The objective of these contractions is to determine the rate of force development.

As recommended by De Luca [49], patients will perform three MVC interspersed by a two-minute rest and the mean of the three contractions will be calculated. Each MVC has to be reached progressively in less than 3 sec and least 5 sec [50]. A five-minute break is scheduled after the MVC because the subsequent part of the trial is the beginning of the fatigability protocol.

The fatigability assessment phase begins with nine contractions at 40% of MVC [51], each lasting 3 sec, followed by an FSC and an MVC. All the contractions are exerted at two-second intervals. This cycle of eleven contractions will be repeated six times. If a patient cannot maintain the 40% required force, the threshold time will be recorded without stopping the test. At the end of these cycles, the patient will rest for 1 min. Then, in order to assess their recovery capacities, the last part of the test will be administered, consisting in three series of one FSC and one MVC, separated by two- and three-minute intervals, respectively.

### Statistical analysis

Sample size calculation was based on the results of the handgrip strength test of Lin et al. [52], who analyzed a relatively large cohort of elderly patients suffering from CKD in 2019, and the handgrip strength reference values of Ramírez-Vélez et al. [36], who analyzed a larger population of elderly patients without CKD, stratified by age and sex. With an 8% (±16%) difference, accepting a 5% alpha risk, 96% statistical power will be reached by enrolling 110 CKD patients and 110 control subjects. Considering the number of patients followed in the UIRAV unit (i.e., approximately 180 older than 60), we should be able to enroll the participants within 1 year.

Signal filtering and analysis will be performed with Matlab 2018a v9.4 (The MathWorks Inc., Natick MA, USA) and statistical analysis will be performed with SPSS v14 (IBM Corp., Armonk NY, USA). Group comparison for normally distributed variables will be assessed by means of the analysis of variance (ANOVA) or the unpaired Student T-Test. For non-normally distributed variables the Kruskal-Wallis or the Mann-Whitney Test will be used. In addition, the chronological variables (e.g., evolution of the MVC during the test) will be analyzed by mixed models with fixed effect or ANOVA with repeated measures if the test prerequisites are satisfied (normality, homoscedasticity and sphericity).

Qualitative data will be compared with the Chi^2^ or the McNemar Test.

Correlation analyses will be performed with the Pearson Test. Logistic or multiple regressions will be performed to assess the effect of different variables on the outcomes: age, sex, body mass index, stage of renal disease, diabetes mellitus, MIS and CCI (either continuous or dichotomized) as well as biochemical data. A *p* < 0.05 will be considered statistically significant.

## Discussion

This study aims to quantify the prevalence of force decline in elderly patients affected by advanced CKD and to identify alterations at the level of the neuromuscular junction. The strength of this study is that it will be, to our knowledge, the first study to assess, in this “pre-dialysis” population, the prevalence of dynapenia coupled with an EMG recording to objectivate electromyographic fatigability features. It will also be the first trial combining an objective (i.e., F_crit_) and a subjective (i.e., FACIT-F and MFI-20) approach to assess fatigue in this population. The link between neuromuscular alterations and greater fatigue is in fact poorly understood [16], especially for CKD patients.

A higher prevalence of dynapenia is expected in the CKD population compared to the control group, with the highest prevalence in those with diabetes mellitus. Patients with diabetes appear to have decreased muscle functions [53, 54], but this result has been questioned [55]. We expect different neuromuscular features in CKD patients, specifically for F_crit_. CKD has been associated with difficulty in performing a standardized task but the origin of this disturbance (e.g., difficulty adapting the motor-unit discharge or difficulty recruiting the motor unit) is far less clear [48]. Our study will allow us to investigate these issues and clarify the underlying pathophysiological mechanisms. The temporal difference between the onset of the EMG signal and force production, i.e. the electromechanical delay, will be assessed during the FSC. We expect an increase in this delay, highlighting an alteration between the electrochemical process (i.e., synaptic transmission, propagation of the action potential, excitation-contraction coupling) and contractile and structural elements. A positive correlation is expected between subjective fatigue and objective fatigability.

The frailty of the elderly CKD population demands careful attention to the methodology employed, explaining the choice of this sub-maximum protocol to reduce as far as possible causing pain while inducing sufficient fatigue in this fragile population.

Better comprehension of the neuromuscular features of dynapenia and a deeper understanding of the relationship between fatigability and fatigue can help us guide interventions for elderly patients suffering from advanced CKD, with the final goal of targeting interventions to effectively improve quality of life and reduce morbidity.

## Data Availability

The details, location and accessibility of the results collected during this study will be reported in a peer-reviewed report, referencing this protocol article.

## Abbreviations

ANOVA: ANalysis Of VAriance
CCI: Charlson Comorbidity Index
CKD: Chronic Kidney Disease
CKD-EPI: Chronic Kidney Disease EPIdemiology Collaboration
EMG: ElectroMyoGraphy
FACIT-F: Functional Assessment of Chronic Illness Therapy-Fatigue
Fcrit: critical Force
FSC: Fast Sub-maximum Contraction
GFR: Glomerular Filtration Rate
MFI: Multifactorial Fatigue Inventory
MIS: Malnutrition Inflammation Score
MVC: Maximum Voluntary Contraction
UIRAV: Unité pour l’Insuffisance Rénale AVancée
